# From Looking at the Floor to Looking Forward: A Case of Hyperkyphotic Spine in an Ankylosing Spondylitis Patient

**DOI:** 10.7759/cureus.70091

**Published:** 2024-09-24

**Authors:** Malwin Singh, Mohd Hisam Muhamad Ariffin, Jin Aun Tan, Suffian Sabri, Azmi Baharuddin

**Affiliations:** 1 Orthopaedics and Traumatology, Universiti Kebangsaan Malaysia Medical Centre, Kuala Lumpur, MYS

**Keywords:** ankylosing spondylitis, chin-brow vertical angle, kyphosis, pedicle subtraction osteotomy (pso), sagittal imbalance

## Abstract

Ankylosing spondylitis (AS) is a chronic inflammatory disorder characterized by progressive spinal stiffness and deformity, primarily affecting the sacroiliac joints, spine, and pelvis. In advanced cases, untreated AS can lead to severe kyphosis, resulting in debilitating functional impairment and a significantly reduced quality of life. We present a case of a patient with a fixed thoracolumbar kyphotic deformity that severely affected his daily function. He underwent an L1 and L4 pedicle subtraction osteotomy (PSO) and posterior spinal instrumentation. This case highlights the challenges associated with correcting rigid spinal deformities in AS patients, where surgical intervention is often the only option to restore function and quality of life. Despite the high risk of complications, advancements in surgical techniques and implants have improved outcomes even in the most complex cases. Detailed preoperative planning, precise surgical execution, and cautious postoperative management are crucial for successful outcomes in such high-risk procedures.

## Introduction

Ankylosing spondylitis (AS) is a rare seronegative spondyloarthropathy affecting approximately 1.4% of the general population, with a prevalence ranging from 0.03% to 1.8% [1], more commonly affecting males than females. It is a chronic inflammatory spondyloarthropathy, primarily affecting the sacroiliac joints, pelvis, and spine, characterized by bridging spinal syndesmophyte formation, enthesitis, sacroiliitis, and uveitis.

Untreated AS has severe, debilitating outcomes, including severe kyphosis of the spine and an increased chin-brow vertical angle (CVBA), leading to severe deformity and functional impairment in approximately 30% of patients [2]. The CBVA represents the angle between a line connecting the chin and brow and a vertical plumb line when the patient stands with hips and knees fully straightened. This angle is crucial in assessing the forward gaze and overall alignment, particularly in patients with severe kyphosis. Ankylosing spondylitis not only affects physical well-being but also causes psychological distress due to social limitations [3].

As treating surgeons, our primary goals are to halt the progression of kyphosis, improve fixed sagittal imbalance, restore horizontal gaze, reduce pain, and enhance function so the patient can continue their daily activities and participate in society. In other words, we should not let AS cause dependency.

In the past, outcomes of complex spinal surgeries had high complication rates, including medical, mechanical, and neurological issues; however, with advances in implants and mechanical devices, we are now able to achieve good surgical outcomes, even in elderly patients [4].

This article aims to illustrate the preoperative planning, surgical nuances, and challenges encountered in managing this complex case. In the thoracolumbar spine, one of the main challenges is accurately predicting the postoperative sagittal balance and addressing any residual imbalance [5]. Achieving optimal sagittal alignment is critical for patient outcomes, as it directly impacts both functionality and long-term quality of life. Preoperative assessments, including radiographic analysis, virtual planning, and alignment goals, are key steps in ensuring precise corrections during surgery. However, intraoperative decision-making remains challenging, requiring the surgeon to balance technical execution with the unpredictability of spinal deformities.

## Case presentation

A 51-year-old male patient presented to our center, with a 20-year history of back pain dating back to 2004. The patient reported an insidious onset with gradually worsening back pain, which initially began as mild discomfort but progressively intensified. Over time, the pain started affecting his daily activities, causing significant discomfort while walking, sitting, and performing routine tasks.

**Figure 1:**
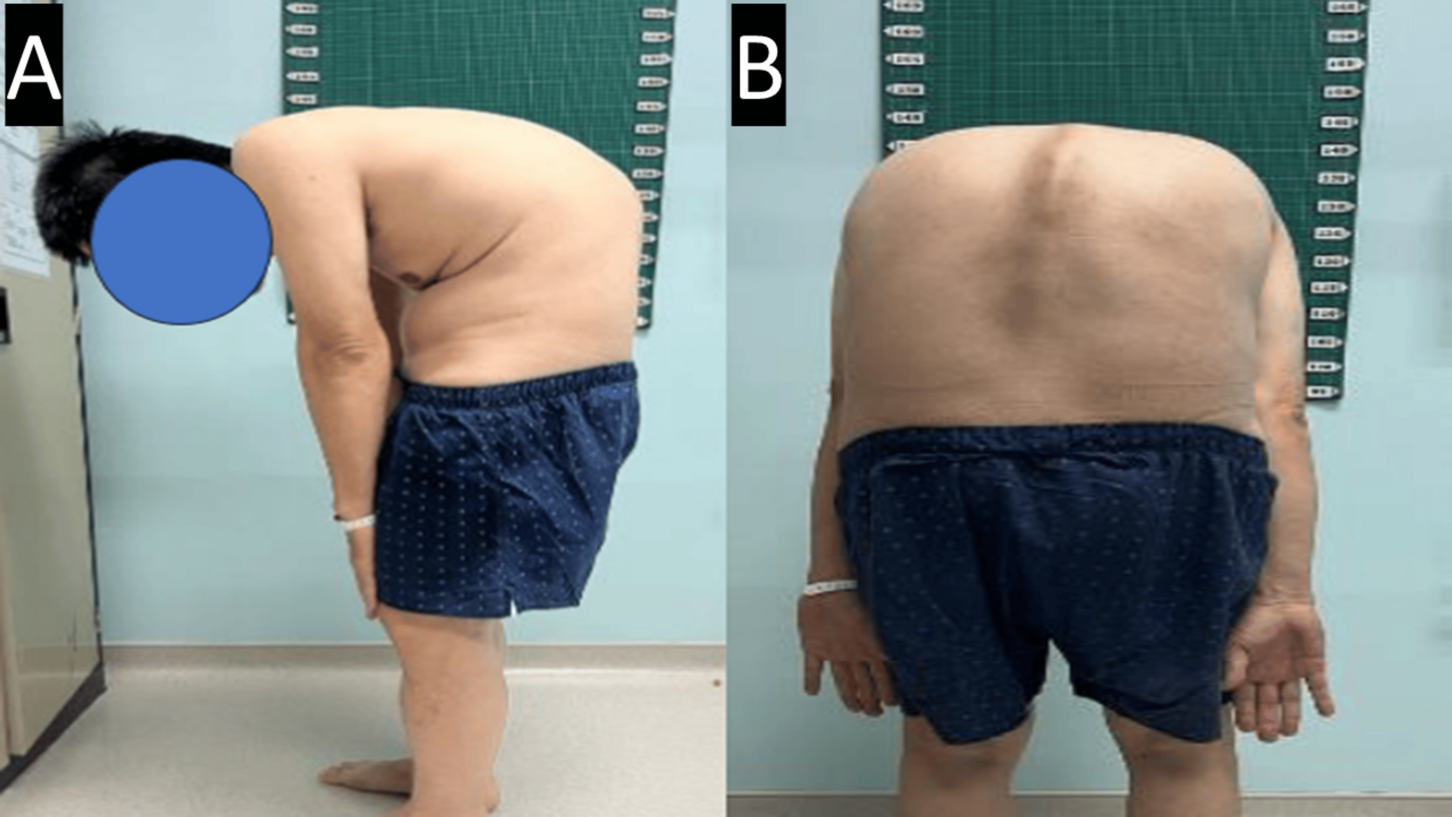
Clinical picture of the patient taken preoperatively. The chin-brow vertical angle is 88 degrees. Figure 1A: A side-ways photo of the patient obtained to show the amount of kyphosis clinically the patient has. Figure 1B: Back view of the patient to show the amount of kyphosis clinically the patient has.

Approximately 15 years ago, he consulted an orthopedic surgeon and was offered surgical intervention. However, the patient declined surgery at the time, as he was still able to perform his job and the spinal deformity was not severe. Despite ongoing pain, he chose to manage his condition conservatively, only with analgesics. Over the years, the deformity progressively worsened until he was no longer able to maintain a forward gaze and was unable to ambulate properly, which led him to seek further medical attention. Otherwise, he had no history of surgical intervention and no other medical illnesses. Figure 1 shows the patient's deformity.

Physical examination revealed a severely deformed spine, appearing stooped forward. There was a loss of lumbar lordosis with hyperkyphosis at the thoracic level, along with a forward head posture and anterior chest wall collapse. Upon ambulation, he exhibited a shuffling gait. It was also noted that he had a fixed flexion deformity of the right hip at 10 degrees but no fixed flexion deformity of the left hip. Using the American Spinal Injury Association (ASIA) assessment protocol, our examination revealed no signs of motor weakness or hypoesthesia in the lower extremities, classified as ASIA grade E. The Oswestry Disability Index (ODI) score indicated severe disability.

**Figure 2:**
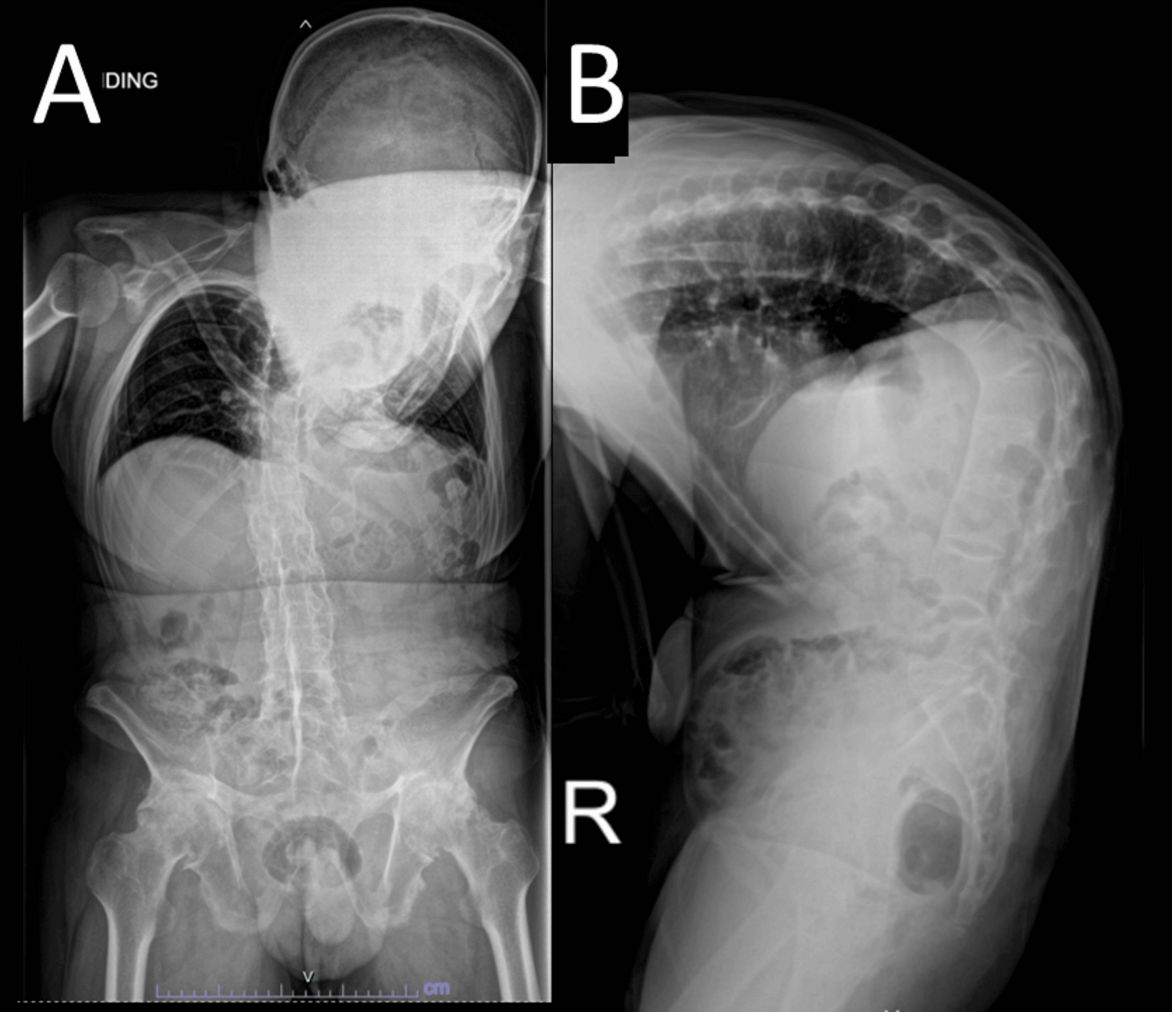
Spinal X-ray before operation Figure 2A: X-ray (anteroposterior view) of the whole spine of the patient; Figure 2B: X-ray (lateral view) of the whole spine of the patient.

**Figure 3:**
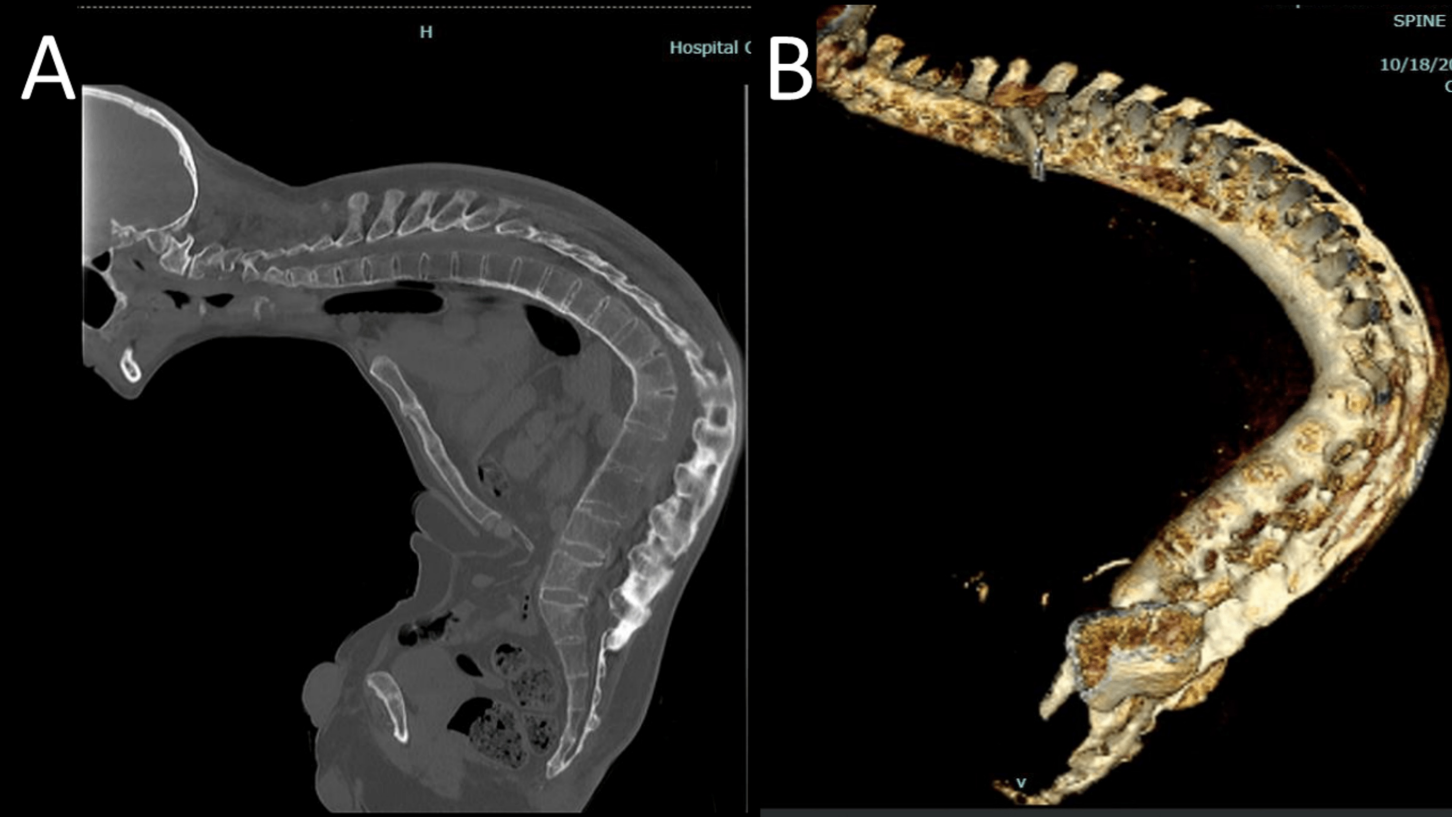
Spinal CT obtained before operation Figure 3A: A CT scan (sagittal view) of the patient showing an ankylosed spine; Figure 3B: Three-dimensional reconstructions of CT scan of the ankylosed spine of the patient

Radiographic examination, as shown in Figure 2, revealed an ankylosed spine (bamboo spine), with loss of lordosis over the lumbar and cervical regions, and an 85-degree kyphotic angle in the thoracic region. The sagittal vertical axis (SVA) was around 68 mm. The sacral slope value was 33 degrees, the pelvic tilt was 5 degrees, and the pelvic incidence was 38 degrees. A computed tomography (CT) scan was also performed for preoperative planning, as shown in Figure 3.

The patient underwent elective surgery after a three- to five-day preoperative period. The procedure was performed under general anesthesia with the patient in a prone position, and the eyes were protected given the length of the surgery. The patient was intubated without any difficulties. We used a Jackson table, allowing us to correct the spinal alignment once the osteotomy was completed. A cervical Mayfield clamp was also applied to ensure the patient's position remained stable throughout the surgery. The surgical area was disinfected with povidone-iodine and draped using a transparent waterproof dressing.

A posterior midline incision was made over the targeted vertebral segment, followed by dissection through the subcutaneous layers to expose the spinous processes. Paraspinal muscles were elevated subperiosteally using an anterograde approach to minimize tissue damage and bleeding. The dissection was extended laterally to reveal the transverse processes and facet joints, providing access to the lumbar spine structures for surgical intervention.

The muscle and tissue behind the vertebral body were carefully stripped, and the position of the preoperatively planned osteotomized vertebra (OV), which is over the L1 and L4, was fluoroscopically located using a C-arm X-ray machine. A pedicle puncture was then performed. The PSO was carried out by removing the lamina of the apical vertebra, followed by decompression of the upper and lower laminae. Under continuous irrigation with normal saline, a grinding drill was used to remove part of the OV through the bilateral pedicles. While preserving the inner wall of the pedicle as much as possible, the drill was used to thin the outer and anterior walls of the vertebral body laterally and anteriorly, creating a channel through the middle of the vertebral body. Finally, the bone of the posterior wall of the vertebral body was removed using tools such as rongeurs and scrapers to create a narrow, wedge-shaped space inside the vertebral body.

After completing the osteotomy, the operative table was adjusted to correct the deformity, and gradual closure of the osteotomized space was observed. A pre-bent titanium rod of appropriate length was used, and the osteotomized end was closed gradually by applying appropriate pressure between the screws. Pedicle screws were inserted from T10 to S1, excluding the L1 and L4 levels. Fluoroscopy with a C-arm X-ray machine was used to confirm the closure of the osteotomized surface and the angle of the osteotomy. The dural sac and the corresponding nerve roots were re-examined to ensure there was no compression or injury.

**Figure 4:**
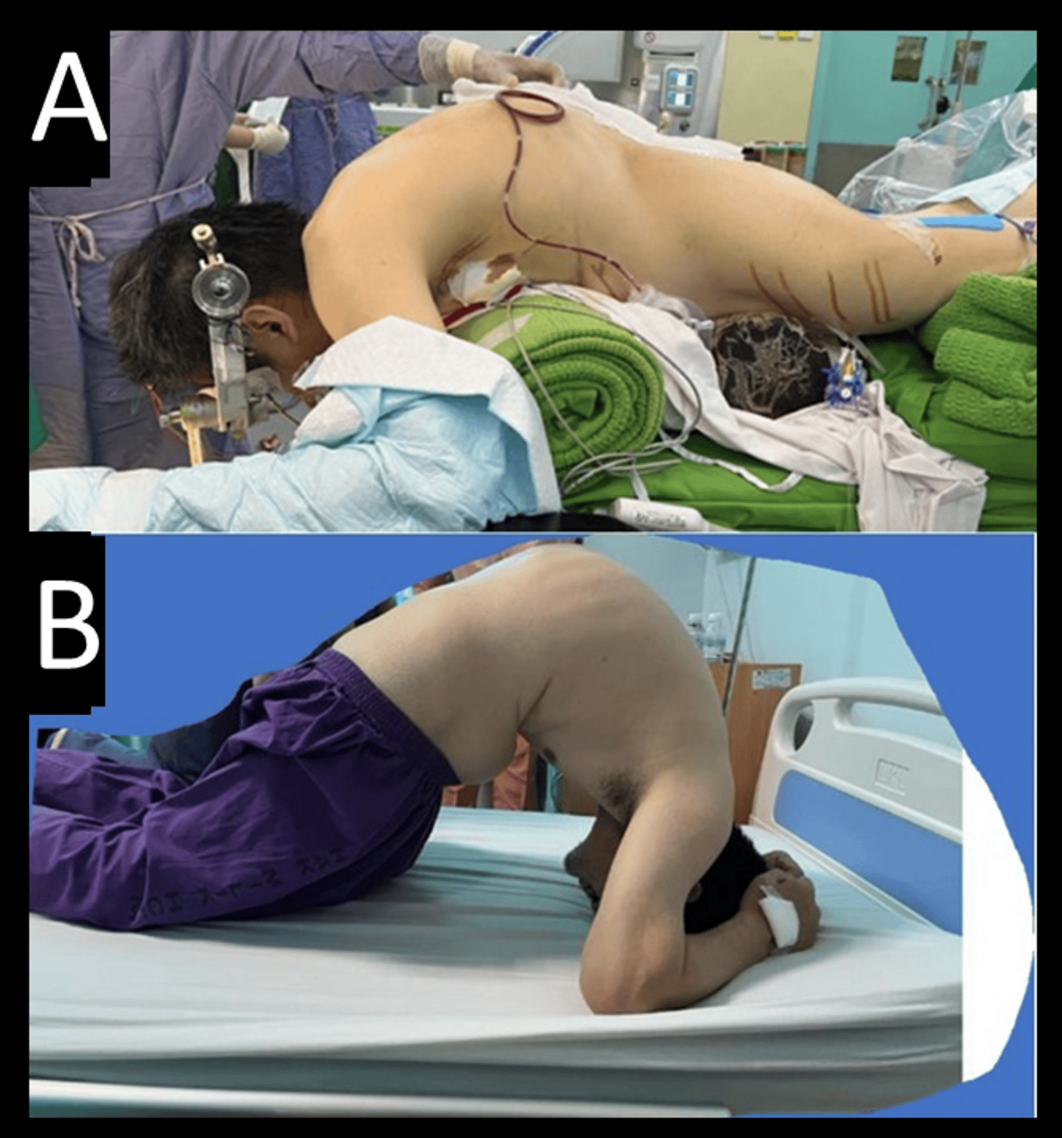
Immediate post- and preoperative difference Figure 4A: Postoperative correction of the spine; Figure 4B: Preoperative clinical deformity of the patient

**Figure 5:**
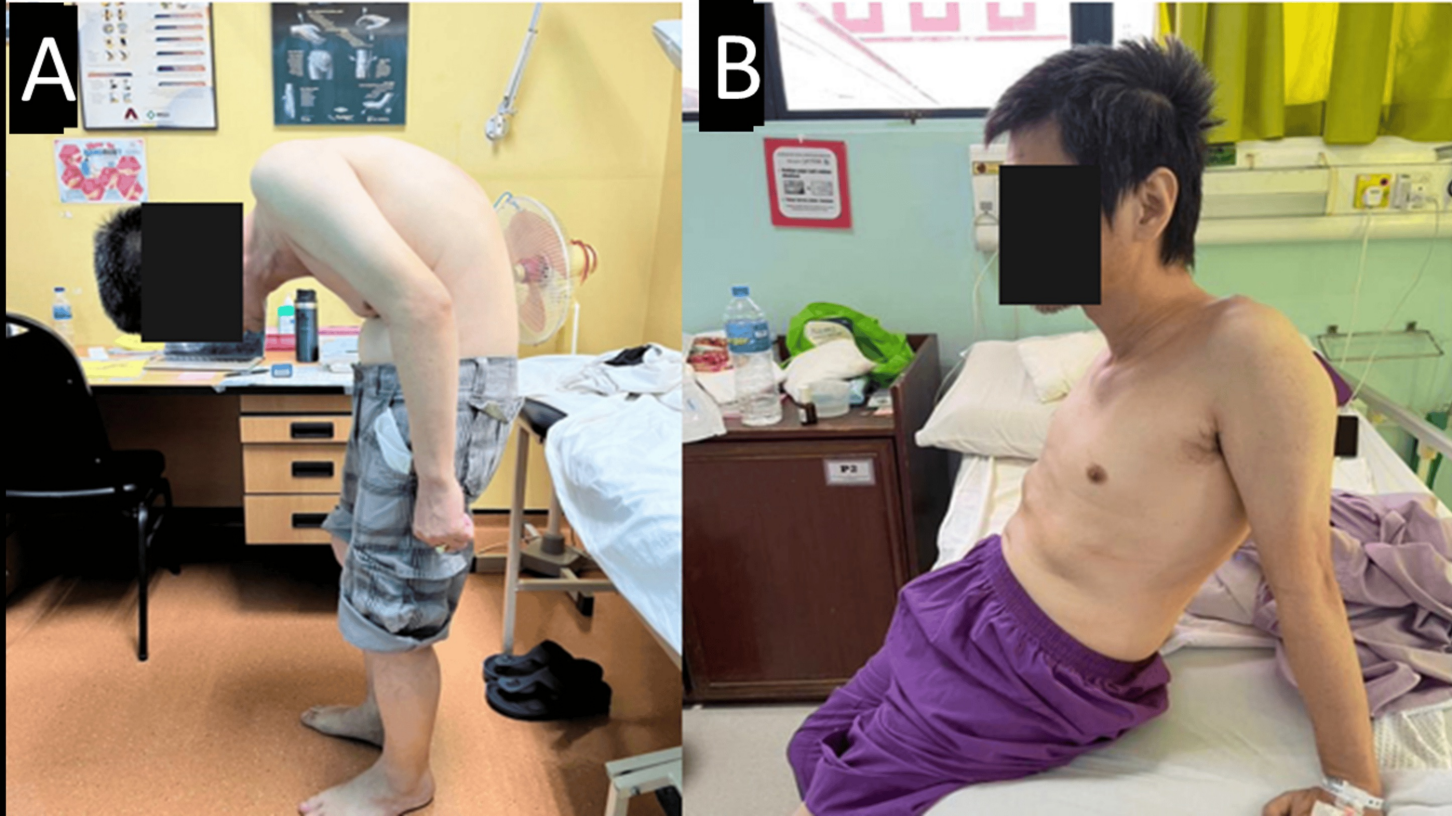
Significant improvement of chin-brow vertical angle (CBVA) from around 88 degrees to 5 degrees is noted Figure 5A: Preoperatively noted CVBA at 88 degrees; Figure 5B: Postoperatively noted CVBA at 5 degrees

A pedicle subtraction osteotomy (PSO) was performed at L1 and L4. During the reduction and correction of the kyphosis, intraoperative neurophysiological monitoring (IOM) was employed to assess neurological function, and no signal changes were detected, indicating preserved neurological integrity. Once optimal spinal alignment was achieved, four rods were used for final fixation. The correction of the hyperkyphosis was observed immediately postoperatively in the operating theater itself, as seen in Figure 4. A drain was placed to manage postoperative bleeding before closing the incision and applying a sterile dressing. Intraoperative blood loss was approximately 800 cm³, necessitating the transfusion of two units of packed red blood cells. No surgical complications were observed. Once the patient was stable and able to sit up, we could observe the amount of correction we achieved, marked by the improvement of the chin-brow vertical angle (CBVA), as seen in Figure 5.

**Figure 6:**
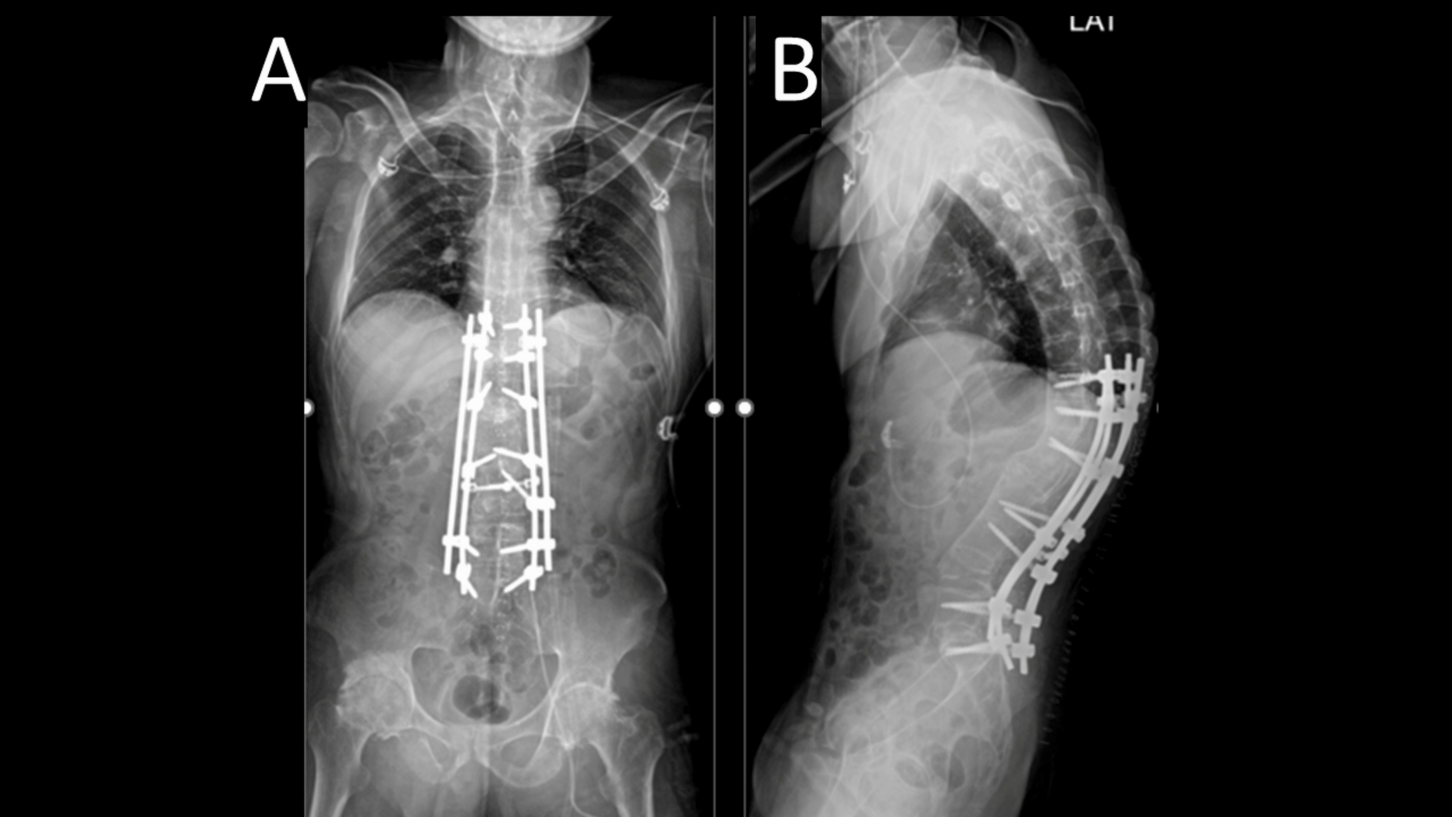
Postoperative X-ray of the patient Figure 6A: Postoperative X-ray (anteroposterior view) showing posterior instrumentation and fusion done from T10-S1; Figure 6B: Postoperative X-ray (lateral view) showing posterior instrumentation and fusion done from T10-S1, showing 70 degrees of kyphosis corrected

Postoperatively, the patient was monitored in the ward for two weeks, with pain management primarily using nonsteroidal anti-inflammatory drugs (NSAIDs). Postoperative X-rays were performed and showed a marked improvement in the kyphosis with 70 degrees of kyphosis corrected, as shown in Figure 6. Rehabilitation was initiated but approached cautiously due to the potential risk of vertebral fractures, given the increased stress on the fixation caused by the newly corrected alignment. The rehabilitation started slowly, initially by sitting on the bed, then to transferring to a wheelchair, and after two weeks, started walking frame ambulation. The surgical drain was removed on day five, and the patient began ambulation shortly thereafter. Rehabilitation was deliberately paced to avoid overloading the vertebrae, which could inadvertently lead to fractures due to the altered biomechanical forces.

## Discussion

Rigid kyphotic deformity or fixed sagittal imbalance presents a significant challenge from a spinal surgeon's perspective. What differentiates corrective surgery in patients with AS from other adult spinal deformities (ASD) is that AS typically results in a stiff, fused spine. This rigidity makes surgical correction more complex, as it limits flexibility and increases the risk of complications during spinal realignment.

There are specific indications for performing osteotomies to correct kyphotic deformities in AS patients. These include severe disruption of sleep or daily activities due to the deformity, compression of internal organs such as the lungs or gastrointestinal system (leading to impaired breathing or digestion), and cases where conservative management fails [5-6], which occurs in most instances. Surgery aims to alleviate these functional impairments and restore a more natural alignment, significantly improving the patient’s quality of life.

The challenge here is determining the type and level of osteotomy to be performed. Each type of spinal column osteotomy offers a different range of correction angles, potential risks, and suitability. A common spinal osteotomy is the Smith-Petersen osteotomy (SPO), also known as a trans-articular process osteotomy. However, this type of osteotomy is not applicable in this case because it only provides about 10 degrees of correction [7] and can only be performed if there is no ossification of the anterior longitudinal ligament [8]. Pedicle subtraction osteotomy involves performing a vertebral body osteotomy using a pedicle to shorten the middle and posterior columns, allowing for a correction of 35-40 degrees at a single level [9].

Vertebral column resection (VCR) is a highly specialized procedure primarily indicated for severe spinal deformities, such as moderate to severe kyphosis, as well as cases involving spinal tumors and complex congenital deformities like hemivertebrae. The procedure involves the complete removal of one or more vertebral segments, allowing for dramatic correction of deformities that cannot be addressed through less invasive techniques. A VCR is especially useful in situations where traditional osteotomies would not provide sufficient correction due to the rigidity or angularity of the deformity [10].

In cases of severe kyphosis with a rigid spine, one of the key factors for successful surgery is proper and detailed preoperative planning. Each clinical practice has its own methods for calculating the osteotomy angle, such as paper-cutting splicing, software analysis, and empirical judgment. In our center, we used both the paper-splicing method and software measurements. However, two key factors that determine the osteotomy plan and angle are the CBVA and sagittal spine imbalance.

Sagittal spine imbalance is assessed by measuring the horizontal distance from the upper back corner of the sacrum to the C7 plumb line on a full-length lateral X-ray taken in a standing position. This measurement indicates the degree of forward or backward displacement in the spine and guides surgical decisions aimed at restoring balance.

In cases of hyperkyphosis, we cannot rely solely on a surgeon’s experience or Cobb's kyphotic angle to determine the angle of osteotomy because every correction directly affects the patient’s CBVA, thus influencing their daily living and work. Performing osteotomies at lower vertebral levels offers greater safety for the same correction angle. Osteotomies in the lumbar region provide a larger operative space and a greater range for correction, allowing the procedure to be carried out safely and with greater accuracy. The first vertebra (L1) is the vertex of the lumbar vertebrae, and osteotomy at this level should achieve better correction. We opted for PSO at the levels of L1 and L4, which allowed for 70 degrees of correction. Since the levels are far apart, the risk of cord buckling was relatively lower, as excessive shortening of two spinal segments may cause flexion of the spinal cord and dura mater [11].

In cases of multilevel osteotomy with high degrees of correction, there are multiple risks, such as nerve injury, prolonged surgery, blood loss, and postoperative complications. To reduce these risks, a comprehensive approach to preoperative planning is essential. Complications associated with lumbar osteotomy primarily include nerve injury, cerebrospinal fluid leakage, incomplete osteotomy closure, iatrogenic fracture, surgical site infection, implant failure, and superior mesenteric artery syndrome. These complications underscore the need for meticulous surgical technique, careful preoperative planning, and vigilant postoperative care to minimize risks and ensure optimal outcomes [12-13].

Multilevel osteotomies and large exposed osteotomy surfaces during surgery result in significantly higher blood oozing, which is the most challenging and time-consuming aspect of the procedure. Subperiosteal dissection and maintaining controlled blood pressure can significantly reduce intraoperative blood loss. For patients experiencing significant blood loss during surgery, autologous blood transfusion is a viable option for managing the blood loss [14].

## Conclusions

Ankylosing spondylitis is a severely debilitating disease that leads to significant functional impairment, affecting patients in all aspects of life. If not managed early, the deformity will progress, leading to more complex surgeries with a higher risk of complications. Early surgery may be considered to avoid more radical interventions. This case also highlights PSO as an effective option for correcting rigid kyphotic deformities in AS when conservative treatments fail. Comprehensive preoperative planning, along with an experienced surgeon, is crucial to ensuring a successful surgical outcome.
